# Valorization of a Byproduct for Sustainable Formulations: Preparation of an Alcohol Gel Hand Sanitizer Containing Glycerol and Methyl Salicylate

**DOI:** 10.1021/acsomega.6c00389

**Published:** 2026-04-17

**Authors:** Ana Michelle Aniceto, Adria T. S. Fernandes, Moises Pedro, Mateus Costa Viana, Guilherme Carneiro, Kelly Cristina kato, David Lee Nelson, Bruno Spinosa De Martinis, Jacques Florêncio, Sandro L. Barbosa

**Affiliations:** 1 Department of Pharmacy, Universidade Federal dos Vales do Jequitinhonha e Mucuri-UFVJM, Campus JK, Rodovia MGT 367 - Km 583, n0 5.000, Alto da Jacuba, Diamantina CEP 39100-000, MG, Brazil; 2 Universidade de São Paulo, Faculdade de Filosofia, Ciências e Letras de Ribeirão Preto, Av. Bandeirantes, 3900, Ribeirão Preto, SP 14040-900, Brazil

## Abstract

Advancements in production techniques have markedly enhanced potential glycerol applications across various sectors. The present study describes the purification of two different samples of crude glycerol obtained from industrial processes. First, the purification of crude glycerol (pH = 5) was achieved by treatment with KOH and subsequent adsorption on commercial activated charcoal. K_2_SO_4_ was also obtained as a byproduct of the process. The purification of crude glycerol (pH = 12) was also achieved by treatment with H_3_PO_4_ and subsequent adsorption on commercial activated charcoal. KH_2_PO_4_ was also obtained as a byproduct of the process. The purified glycerol was analyzed by IR, ^1^H and ^13^C NMR, and the ASTM D4176–22 method (standard test method for free water and particulate contamination). ASTM D874 is the standard test method for determining the sulfated ash content. The test is used to measure the amount of inorganic contaminants (ash-forming materials) such as abrasive solids and catalyst residues that might remain in glycerol after the purification process. These materials often contain metals such as calcium, potassium, and sodium, and ASTM D664 is a standard potentiometric titration method to determine the total acid number in fatty acid methyl esters (biodiesel or biofuel) by measuring free fatty acids and mineral acids, expressed as mg KOH/g; while not directly used for glycerol, it is crucial for determining glycerol quality because crude glycerol from biodiesel production contains impurities such as free fatty acids that ASTM D664 quantifies, helping to determine if glycerol is suitable for further processing. Subsequently, glycerol was applied directly in the formulation of a hand sanitizer. The hand sanitizer was prepared by mixing water, glycerol, hydroxyethylcellulose, and a solution of methylparaben-ethanol. Methyl salicylate was added to the formulation after the homogenization process, and a hand sanitizer without methyl salicylate was prepared for comparison. In the context of ecosustainable formulation design, this study addresses the self-aggregation of glycerol and methyl salicylate with a wide range of physicochemical and biological activities in hand sanitizer. The physicochemical properties of the formulation were determined in a preliminary analysis of the properties of the hand sanitizer. The pH of the hand sanitizers was slightly acidic (5.7), and despite their high viscosities, the formulations had a high degree of spreadability. The aggregation of methyl salicylate was determined at concentrations up to 40–50 wt %. These results provide basic knowledge to promote the exploration of glycerol and methyl salicylate as valuable ingredients in formulations for various applications.

## Graphical abstract

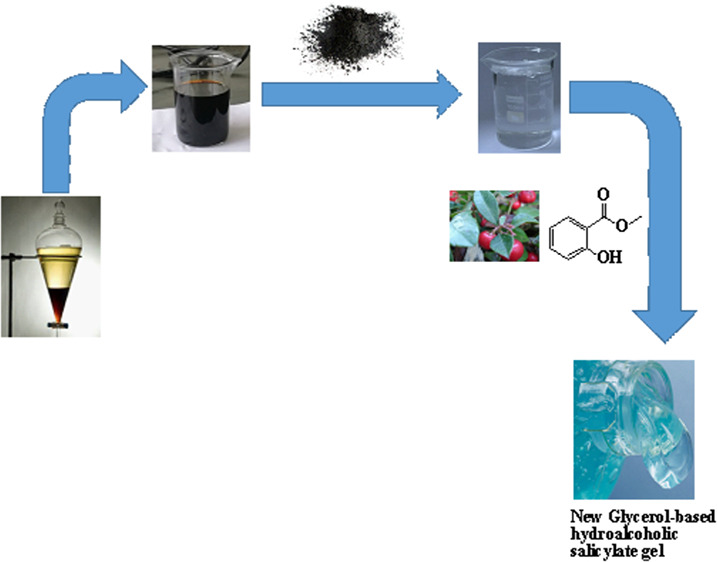

## 1 Introduction

Rising demand for affordable, renewable energy has boosted biodiesel production worldwide, resulting in greater generation of glycerol.^1^ In 2024, the global glycerol market stood at approximately 4.442 million tons, and it was estimated to be around 4.78 million tons in 2025, with a projected growth to 5.95 million tons by 2030.^2^ The glycerol market in Latin America, or more precisely South America, Brazil, and Argentina, is a key player in glycerol production within the region, and the market is experiencing growth, driven by greater biodiesel production and the increased demand from the personal care and cosmetics industries.^3^

Glycerol as byproducts of the transesterification process of triglycerides from vegetable oils.^4,5^ Tan et al.^6^ review the different methods of producing crude glycerol by transesterification or saponification reactions, as well as acid hydrolysis. Glycerol is a trihydroxy propane that possesses great potential applications in the cosmetics industry.^5,6^ It is used because of its soothing effect, and it is also important in the production of syrups, creams, and ointments.^7^ In pharmaceutical formulations, the viscosity of some pharmaceutical preparations, lubrication, and moisture retention or humectant properties are enhanced by the addition of high-purity glycerol, in addition to clinically acting as an antimicrobial and anti-inflammatory agent.^8^ Glycerol can act as a cosolvent, emollient (in hand sanitizers, *inter alia*), humectant, plasticizer, sweetening agent, and tonifying agent.^9^

Glycerol is widely used in many oral formulations, where it acts as a sweetening agent, and in ophthalmic, topical, and parenteral formulations.^10^ Glycerol is a plasticizer in film coatings, gelatin capsules, and suppositories.^11^ Hence, glycerol is susceptible to a variety of chemical transformations to different products.^6,12–14^

Biomass waste generated by the agricultural sector, such as the processing of palm oil in Cameroon and other African countries, can be absorbed by relevant industries as feedstock to extract vegetable oil for green energy, as well as byproducts for the cosmetic, pharmaceutical, and allied industrial uses.^15,16^

The ethanol-based handrub (EBHR) formulation of the World Health Organization (WHO) contains 1.45% glycerol as an emollient to protect the skin of healthcare workers (HCWs) against dryness and dermatitis. The minimal concentration of glycerol required to protect hands was unknown. However, glycerol seems to negatively affect the antimicrobial efficacy of alcohols. Meneguet et al.^17^ evaluated the tolerance of HCWs to the WHO EBHR formulation using different concentrations of glycerol in a tropical climate healthcare setting. A randomized cluster, double-blind, crossover study among 40 HCWs from an intensive care unit of a tertiary-care hospital in Brazil was conducted from June first to September 30, 2017. The original WHO EBHR formulation containing 1.45% glycerol was compared with three other concentrations (0, 0.5, and 0.75%) of glycerol. A generalized estimating equation of the logit type was employed to compare differences among the tolerabilities with different formulations. Participants had 2.4 times (95%CI: 1.12–5.15) greater chance of having a skin condition considered to be good when they used the alcohol containing 0.5% glycerol instead of the 1.45% glycerol formulation. For the self-evaluation scale, the values were generally lower (OR: 0.23, 95%CI: 0.11–0.49) for the preparation without glycerol than for the WHO standard formulation (1.45%), and there were no differences between the other formulations used. Thus, the use of the formulation containing 0.5% glycerol led to the best ratings of skin tolerance.

Glycerol from the biodiesel industry can contain large amounts of impurities, such as soaps, MeOH, FAME, mono-, di-, and triglycerides, water, and other organic matter, which depend on the type of oil feedstock used, the efficiency of the overall production process, and the separation techniques used at the end of the manufacturing process.^18^ The demand for it in the future might be slightly higher because of economic growth. Thus, new methods of glycerol production, purification, recycling, and utilization are needed.

Dhabhai et al.^19^ and Wan Isahak et al.^20^ described the procedures involved in generating and collecting crude glycerol produced through the transesterification of various triglycerides, and they discussed the technical, economic, environmental, and other considerations associated with purifying crude glycerol for recycling and reuse. Studies of the collection, treatment, and purification methods have shown that these processes are feasible and economically viable.^21–23^ Abdul Raman et al.^24^ used acidification and ion exchange to decontaminate crude glycerol obtained from FAME synthesis, demonstrating its technical, economic, and ecological sustainability. In recent times, the use of other purification techniques, such as electrocatalytic oxidation,^25^ solvent extraction,^26^ distillation,^27^ membrane filtration,^28^ and vacuum evaporation,^29^ has been exploited (Scheme 1).

**Scheme 1:**
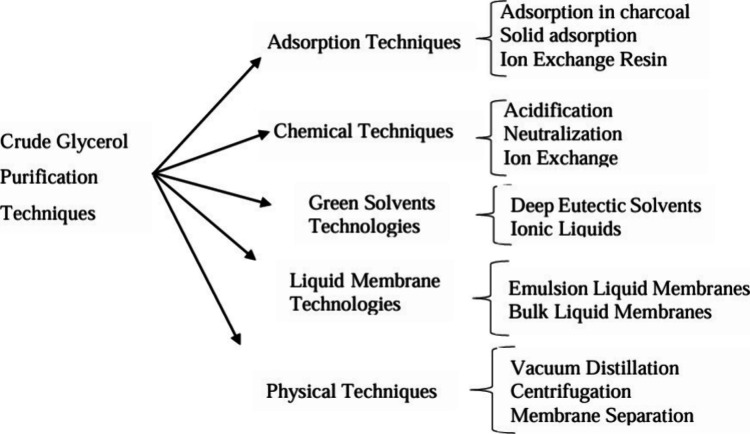
Crude Glycerol Purification Techniques

Recent reviews have examined trends and technologies in the recovery, refinement, conversion, and utilization of crude glycerol.^30,31^ Studies highlight the benefits of purifying crude glycerol and call for research into safe and affordable biotechnological purification methods, improved reaction engineering, and robust kinetic and thermodynamic models. Further advances are recommended in developing eco-friendly catalysts, applying optimization to reduce costs, and scaling up processes to industrial levels.^32,33^

In a recent study, we described the application of glycerol derived from the transesterification reaction of triglycerides catalyzed by inorganic bases, such as KOH, and subsequently purified^34^ for the production of hand sanitizer.^35^ The present work sought to describe a new biotechnological method of purification of two samples of crude glycerol (pH = 5 and 12) generated in the industrial transesterification of various triglycerides to form FAME. We report the purification of industrial crude glycerol obtained from the biodiesel industry and its use, together with methyl salicylate, previously synthesized,^36,37^ in the formulation of a hand sanitizer.

## 2 Experimental Section

### 2.1 Raw Materials and Chemicals

All the reagents were HPLC grade.^35^ Deionized water was obtained using a Milli-DI water purification system from Merck. Ultrapure water was used in the blank gel matrix.^37^ Hydroxyethyl cellulose was obtained from Vetec (São Paulo, Brazil); methylparaben was obtained from EMFAL (Betim, Brazil), and two different samples of crude glycerol (pH = 4 and 12) were obtained from the biodiesel industry. Merck activated charcoal is a porous, carbon-rich residue created through pyrolysis (heating in low-oxygen environments) of biomass materials. Merck charcoal is an amorphous form of carbon with high porosity and a large surface area, typically used for adsorption, purification, and decolorization. Often used for decolorizing (−100 mesh), according to the general industry, with specific products having median diameters around 34 μm.

### 2.2 Purification of the Industrial Crude Glycerol

Purification of crude industrial glycerol was performed using a combination of chemical and physical processes. A 1000 mL portion of crude glycerol (pH = 5) was initially heated at 60 °C to remove excess MeOH used in the transesterification reaction that gave rise to glycerol. The product mixture was washed with three 50 mL portions of hexane to remove the FAME. The glycerol was neutralized using KOH to produce a K_2_SO_4_ precipitate. After decanting for 12 h, the supernatant was filtered, and K_2_SO_4_ was purified by recrystallization from water and stored. The pretreated glycerol was filtered three times through a sintered glass funnel using 10 g of commercial charcoal for each filtration. The glycerol obtained was 99.428% pure according to the method of ASTM D6584. The regeneration of used commercial charcoal was performed after shaking at 250 rpm with MeOH in the ratio of 3:1 (v/w) for 1 h to remove the adsorbed glycerol, and heating in a muffle furnace at 150 °C for 5 h for reactivation. After using the charcoal three times, it no longer retained all of the pigments and required reactivation by heating.

#### 2.2.1 Purification of the Industrial Crude Glycerol pH = 12

A 1000 mL portion of crude glycerol (pH = 12) was initially heated at 60 °C to remove excess MeOH from the transesterification reaction that gave rise to glycerol. The product mixture was washed with three 50 mL portions of hexane to remove the FAME. The glycerol was neutralized using H_3_PO_4_ to produce the KH_2_PO_4_ precipitate. After decanting for 12 h, the supernatant was filtered, and KH_2_PO_4_ was purified by recrystallization from water and stored. The pretreated glycerol was filtered two times through a sintered glass funnel using 10 g of commercial charcoal for each filtration. The glycerol obtained was 99,65% pure according to the method of the ASTM D6584. The regeneration of used commercial charcoal was performed after shaking at 250 rpm with MeOH in the ratio of 3:1 (v/w) for 1 h to remove the adsorbed glycerol, and heating in a muffle furnace at 150 °C for 5 h for reactivation. After using the charcoal five times, it no longer retained all of the pigments and required reactivation by heating.

### 2.3 Preparation of the Alcohol Gel Hand Sanitizer

In a beaker, 30 mL of distilled water was mixed with 3.0003 g of pure glycerol and heated to 60 °C with magnetic stirring. While the mixture was being stirred, 2.0001 g of hydroxyethylcellulose was slowly added to the aqueous phase and allowed to hydrate. Separately, 0.1501 g of methylparaben was dissolved in 90.0 mL of EtOH. After the gel base was cooled to a temperature below 40 °C, the alcoholic solution containing methylparaben was gradually added to the aqueous gel phase with continuous stirring. This alcohol gel hand sanitizer was coded as HS1. In addition, 1.0008 g of methyl salicylate was incorporated into the final product (coded HS2). Both gels were stored in transparent polypropylene bottles with a capacity of 100.00 g.

### 2.4 Characterization of the Hand Sanitizers

#### 2.4.1 pH Measurement

Samples were diluted to a 10% (w/v) concentration, and pH measurements were performed directly using a pH meter (model mPA210, MS Tecnopon Instrumentação, Porto Alegre, Brazil), in triplicate.^38^

#### 2.4.2 Relative Density

The relative density was determined using a clean, dry 25 mL pycnometer. The pycnometer was weighed empty, then filled with water and subsequently with the sample, all maintained at 25 °C. The relative density was calculated as the ratio of the net weight of the sample to the net weight of water.^39^

#### 2.4.3 Apparent Viscosity

The apparent viscosity was measured using a microprocessed rotational viscometer (QUIMIS Q860M21). The appropriate spindle was selected on the basis of the viscosity of the sample and in accordance with the quality control guidelines for hand sanitizing gels established by RDC No. 46, February 20, 2002. Thus, spindle No. 4 at a rotation speed of 20 rpm was used. The spindle was vertically immersed in the sample at room temperature (25 °C), ensuring the absence of air bubbles. Viscosity measurements were then performed in triplicate, following the device’s standard operating procedure.^40^

#### 2.4.4 *In Vitro* Spreadability

The *in vitro* spreadability test was performed following the method described by Knorst (1991),^38^ with modifications. A circular glass mold plate (20 cm in diameter, 0.2 mm thick) was placed on a square glass support plate (20 × 20 cm), with a sheet of graph paper positioned underneath. The sample was introduced into the central opening of the mold and leveled with a spatula. The mold plate was then carefully removed. A preweighed acrylic plate (5 g) was gently placed over the sample. After one min, the spread area was determined by measuring the diameter in two perpendicular directions and calculating the mean. Spreadability (*S*), measured at 25 °C, was calculated using the following equation:

$$S=\frac{\pi xd^{2}}{4}$$

where *S* is the *in vitro* spreadability (mm^2^) of the sample under a weight of 5 g, and *d* is the mean diameter (mm).

#### 2.4.5 Ethanol Concentration

The concentration of ethanol was measured on an Agilent 7890A chromatograph (Agilent Technologies Inc., Santa Clara, CA, USA (GC/FID system) using an Agilent GC autosampler 80 (Agilent Technologies Inc.) as described in the production of the hand sanitizer.^35^

### 2.5 Statistical Analysis

The results were represented as mean value ± standard deviation from determinations in triplicate. The student *t* test was used to analyze the statistical differences between the mean values (α = 0.05).

## 3 Results and Discussion

This work is divided into two parts: the first deals with the development of a process for purifying crude industrial glycerol, and the second involves the use of purified glycerol in the process of formulating alcohol-based hand sanitizer gel.

### 3.1 Purification and Characterization of Crude Industrial Glycerol pH = 5

Crude industrial glycerol had the following characteristics before being purified: total glycerol (ASTM D6584) 70%, humidity (Water by Karl Fischer – ASTM D6304) 23%, monoglyceride index (esters in biodiesel – EN 14103 – adapted) 4%, and pH = 5 at 25.7 °C. After pretreatment using KOH and purification by adsorption using commercial charcoal, the glycerol had the following specifications: total glycerol (ASTM D6584) 99.428%, humidity (ASTM D6304) 0.43%, monoglyceride index (EN 14103) 0.10%, and pH 6.5 at 25.7 °C (Table 1).

**Table 1:** Physical–Chemical Characteristics of Industrial Crude Oil pH = 5 and Purified Glycerol

parameters	industrial crude glycerol	purified glycerol
pH	5.00 ± 0.01	6.50 ± 0.05
relative density (g/cm^3^)	1.200	1.238
apparent viscosity (cP)	27,180 ± 60	27,080 ± 35
total glycerol	70	99.428

#### 3.1.1 Purification and Characterization of Crude Industrial Glycerol, pH = 12

Crude glycerol from the biodiesel industry had the following characteristics before being purified: total glycerol (ASTM D6584) 84%, humidity (Water by Karl Fischer - ASTM D6304) 14%, methanol 1.5%, monoglyceride index (esters in biodiesel – EN 14103 – adapted) 4%, and pH = 5 at 25.7 °C. After pretreatment using H_3_PO_4_ and purification by adsorption using commercial charcoal, the glycerol had the following specifications: total glycerol (ASTM D6584) 99.428%, humidity (ASTM D6304) 0.54%, monoglyceride index (EN 14103) 0.00%, and pH 6.5 at 25.7 °C (Table 2).

**Table 2:** Physical–Chemical Characteristics of Industrial Crude Oil at pH = 4 and Purified Glycerol

parameters	industrial crude glycerol	purified glycerol
pH	12.0 ± 0.01	6.50 ± 0.05
relative density (g/cm^3^)	1.208	1.238
apparent viscosity (cP)	26,198 ± 54	27,080 ± 35
total glycerol	84	99.624

#### 3.1.2 Characterization of Pure Glycerol by ^1^H and ^13^C NMR

The ^1^H NMR spectrum of the glycerol molecule typically showed three main signals, a complex multiplet around 3.5 ppm for the methine proton (CH), and a broader signal for the hydroxyl protons (−OH), around 4.8 ppm, often overlapping the methylene signal (Figure 1).

**Figure 1:**
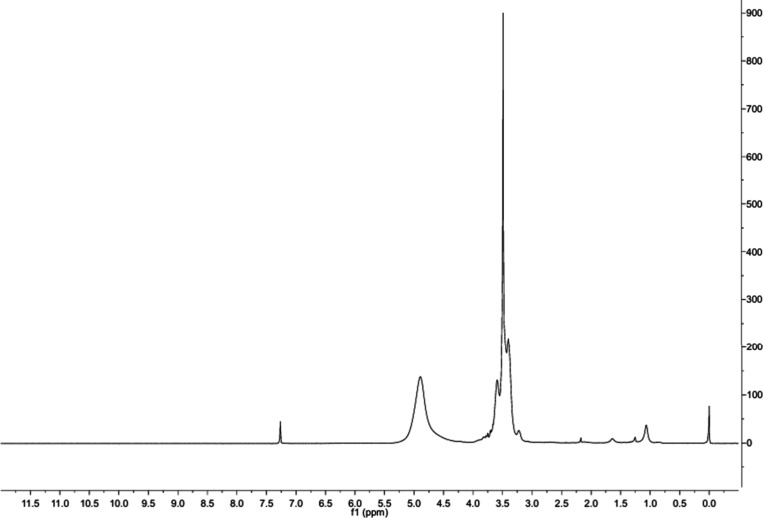
^1^H NMR spectrum (400 MHz, CDCl_3_ as solvent) of purified glycerol is in accordance with the literature.^34,35^

The ^1^H NMR chemical shift and splitting are summarized in Table 3.

**Table 3:** ^1^H NMR Features of the Glycerol Molecule

protons	Chemical shift	Splitting
methylene hydrogens (CH_2_)	∼3.5 ppm	a double doublet (*J* = 4 Hz) centered at 3.5 ppm and a double doublet (*J* = 4 Hz) between 3.54 and 3.60 ppm correspond to the methylene hydrogens
hydroxyl hydrogens (−OH)	4.8 ppm	∼4.8 ppm in CDCl_3_
methine hydrogen (CH)	3.62 to 3.68 ppm	multiplet

The glycerol molecule has a plane of symmetry through the central carbon (C2) that makes the two terminal carbons (C1 and C3) chemically equivalent. The ^13^C NMR spectrum of the glycerol molecule, Figure 2, typically exhibits two signals: a methine carbon (CH) signal around 72.3 ppm and two equivalent methylene carbons (CH_2_) signals around 63.0 ppm, which is in accordance with the literature.^34^

**Figure 2:**
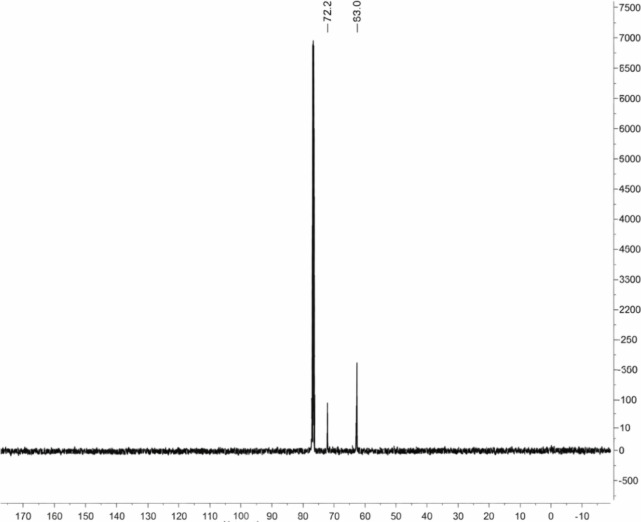
^13^C NMR spectrum (100 MHz, CDCl_3_ as the solvent) of purified glycerol.

#### 3.1.3 Characterization of Pure Glycerol by Infrared (IR)

The IR spectrum of glycerol, is in accordance with literature,^43^ showed characteristic peaks for its hydroxyl (−OH) and C–H bonds, with a broad O–H stretching band around 3200–3400 cm^–1^ (due to strong hydrogen bonding), C–H stretching near 2810–2950 cm^–1^, and C–O stretching and C–O–H bending in the fingerprint region (around 1000–1400 cm^–1^), Figure 3.

**Figure 3:**
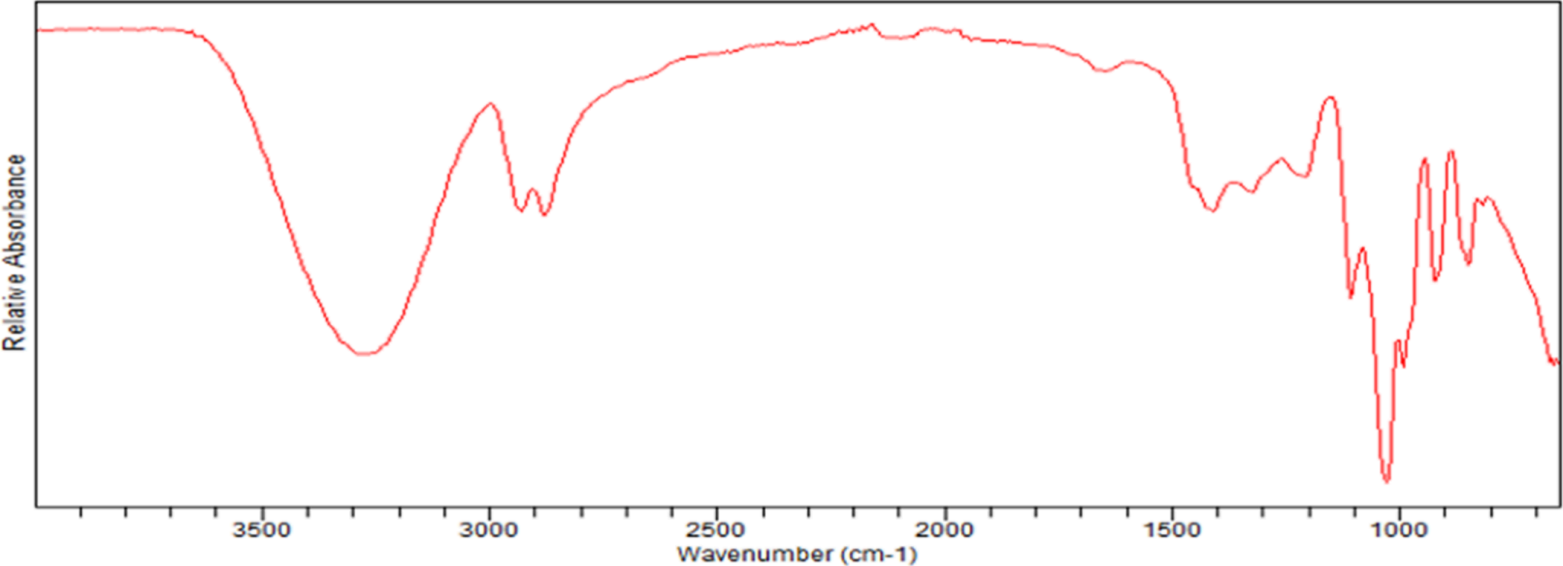
IR spectrum of purified glycerol.

Absorption bands, such as the O–H stretching, are represented by a broad, strong band around 3286 cm^–1^ that indicates extensive hydrogen bonding between glycerol molecules. Aliphatic C–H stretches appear in the 2931 and 2877 cm^–1^ regions; C–O stretching, represented by a prominent band around 1033 cm^–1^, is characteristic of the primary alcohol C–O bond; C–O–H bending. A band near 1400–1420 cm^–1^ is attributed to the bending motion of the C–O–H group (Figure 3).

#### 3.1.4 Characterization of Pure Glycerol by Gas Chromatography and Mass Spectrometry (GC/MS)

Total ion chromatography (TIC) for glycerol, Figure 4, typically obtained via GC-MS, showed its presence as a peak with a retention time of 7.890 min.

**Figure 4:**
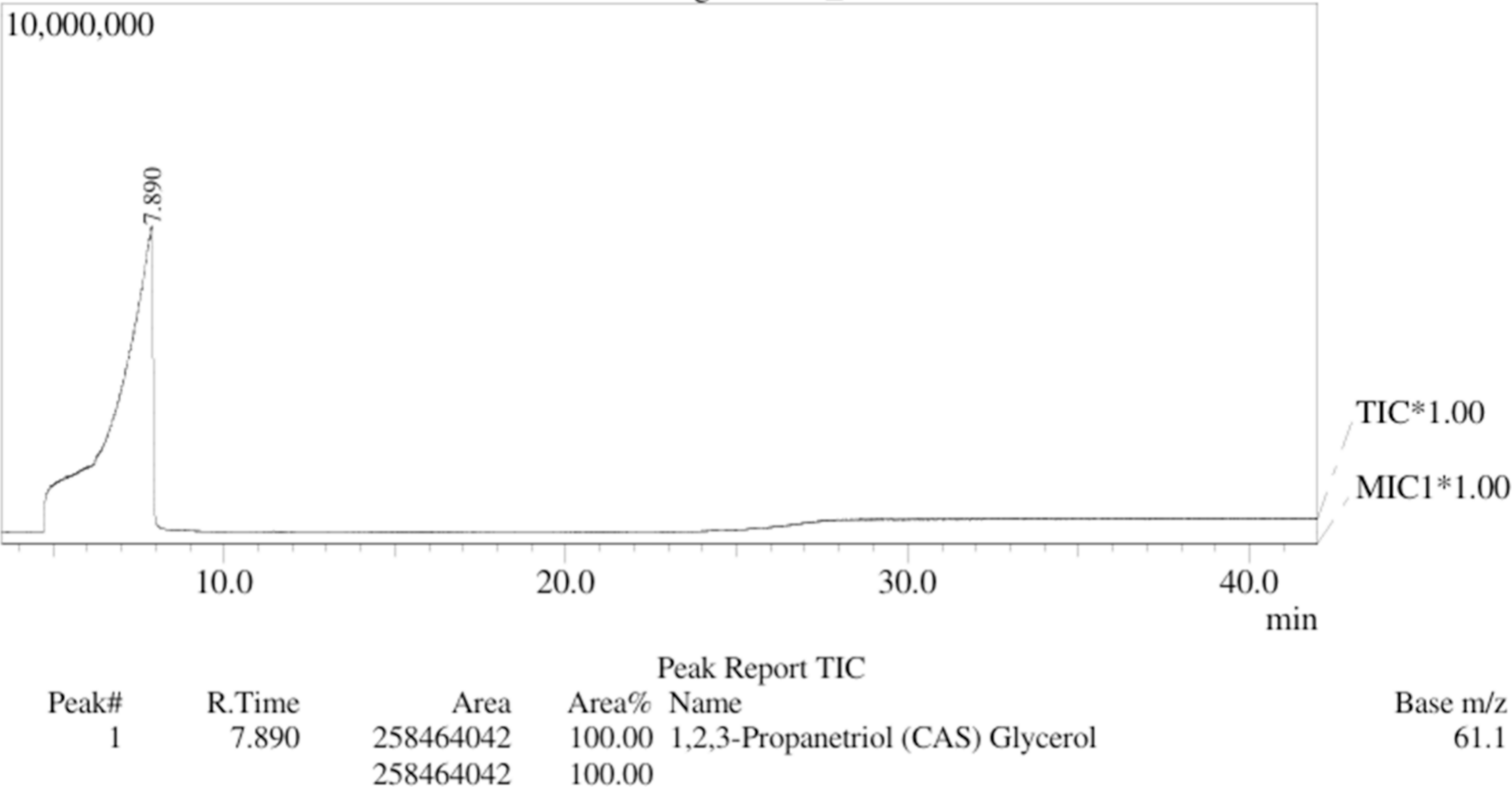
Total ion chromatogram (TIC) for glycerol.

Mass spectrum showing the fragmentation of the glycerol molecule using electron ionization (EI) showed ions such as *m*/*z* 93 (molecular ion, *m*/*z* 75, *m*/*z* 61, *m*/*z* 57, *m*/*z* 45, *m*/*z* 31, and *m*/*z* 29, resulting from sequential losses of water (18 Da) and carbon units, often involving ring structures like dioxolanes (*m*/*z* 75) and hydroxycarbenium ions (*m*/*z* 57, 31). The primary fragmentation pathways involve dehydration and C–C bond cleavage, with ions such as *m*/*z* 75 and *m*/*z* 45 being prominent, alongside characteristic small ions such as *m*/*z* 31 and CHO (*m*/*z* 29), Figure 5.

**Figure 5:**
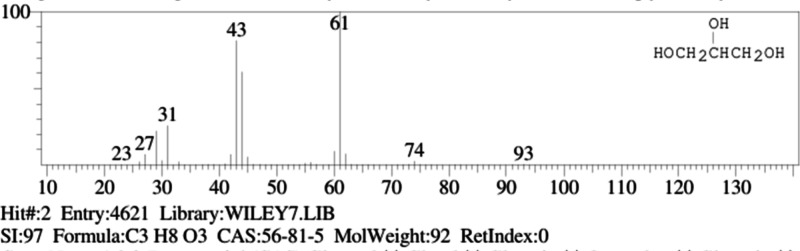
Mass spectrum fragmentation of the glycerol molecule.

Fragments formed by the electronic impact and mechanisms of fragmentation (EI-MS) for glycerol are summarized in Table 4.

**Table 4:** Fragments and Mechanisms (EI-MS) for glycerol

fragments	mechanisms (EI-MS):
*m*/*z* 93 [C_3_H_8_O_3_]^+^	protonated or radical molecular ion
*m*/*z* 75 [C_3_H_7_O_2_]^+^	formed by loss of one water molecule (18 Da) from *m*/*z* 93, often via cyclization to a protonated dioxolane ring
*m*/*z* 61 [(C_2_H_5_O_2_)] ^+^ or (C_3_H_5_O^+^)	further dehydration or fragmentation from *m*/*z* 75 or direct fragmentation
*m*/*z* 57 (C_3_H_5_O)^+^	stable hydroxycarbenium ion, a common fragment
*m*/*z* 45 (C_2_H_5_O)^+^	significant fragment, often related to CH_2_=C(OH)_2_^+^ or similar structures
*m*/*z* 31 CH_2_OH^+^	common fragment from primary alcohol groups
*m*/*z* 29 (CHO^+^ or C_2_H_5_^+^)	very small, abundant ions from the carbon backbone

#### 3.1.5 Characterization of Pure Glycerol from Crude Industrial Glycerol pH = 5

Glycerol's analysis was based on the second ASTM, EN, and NBR, and these are summarized in Table 5.

**Table 5:** Analysis According to the ASTM, EN, and NBR Methods for the Glycerol Compound

essay/method	unit	results
total glycerols - ASTM D6584 (adapted)	% w/w	99.428
water by Karl Fischer - ASTM D6304	% w/w	0.43
esters in biodiesel – EN 14103 (adapted)	% w/w	0.10
density at room temperature - Balloon or pycnometer method	g/mL	1.238
pH - NBR 10891 (10% w/v in water)		6.5 in 25.7 °C
Acidity - ASTM D664 (TAN)	mgKOH/g	0,08
Solubility in water (10% w/v in water)		soluble

#### 3.1.6 Characterization of Pure Glycerol from Crude Industrial Glycerol pH = 12

Purified glycerol's analyses according to ASTM, EN, and NBR are summarized in Table 6.

**Table 6:** Analysis According to ASTM, EN, and NBR for the Glycerol Compound

essay/method	unit	results
total glycerol - ASTM D6584 (adapted)	% w/w	99.654
water by Karl Fischer - ASTM D6304	% w/w	0.54
esters in biodiesel – EN 14103 (adapted)	% w/w	0.0
density at room temperature - balloon or pycnometer method	g/mL	1.238
pH - NBR 10891 (10% w/v in water)		6.5 in 25.7 °C
acidity - ASTM D664 (TAN)	mgKOH/g	0,09
solubility in water (10% w/v in water)		soluble

### 3.2 Ethanol Concentration

The ethanol concentration in all of the samples was determined by GC/FID. Two previously prepared products were also analyzed. All of the samples contained ethanol concentrations equal to or greater than the concentration considered to be effective (70–80%). A simple, rapid HS-GC/FID method for quantifying ethanol in ethanol-based gel hand sanitizers was developed, validated, and applied to the samples. Four samples were analyzed (two without methyl salicylate and two with). The results of the analysis are shown in Table 7.

**Table 7:** Ethanol Concentrations in Samples of Hand Sanitizer with (HS2 and HS2′) and without (HS1 and HS1′) the Addition of Methyl Salicylate

sample	concentration (%)
HS1	74.04
HS1′	73.51
HS2	64.03
HS2′	66.87

### 3.3 Characterization of the Gel Alcohol Hand Sanitizer with (HS2) and without (HS1) Methyl Salicylate

The self-aggregation of glycerol and its interaction with methyl salicylate involve complex hydrogen-bonding networks, often utilized in creating stable formulations, such as nanoemulsions or gels. Glycerol forms strong, extensive hydrogen-bonding networks. In mixtures (such as those with water or solvents), glycerol molecules can form elongated micellar aggregates driven by their hydrogen-bonding capability. In its pure liquid state, methyl salicylate exhibits strong intramolecular hydrogen bonding between its hydroxyl hydrogen and carbonyl oxygen. When mixed, the interactions are primarily noncovalent. While methyl salicylate relies on internal bonding, its interaction with solvents, like glycerol or in micellar systems, involves weak intermolecular hydrogen bonding and van der Waals interactions.^44–46^ When methyl salicylate is incorporated into nanoemulsions, it forms small, stable droplets, where the methyl salicylate molecules are organized within the carrier structure (Chart 1).

**Chart 1:**
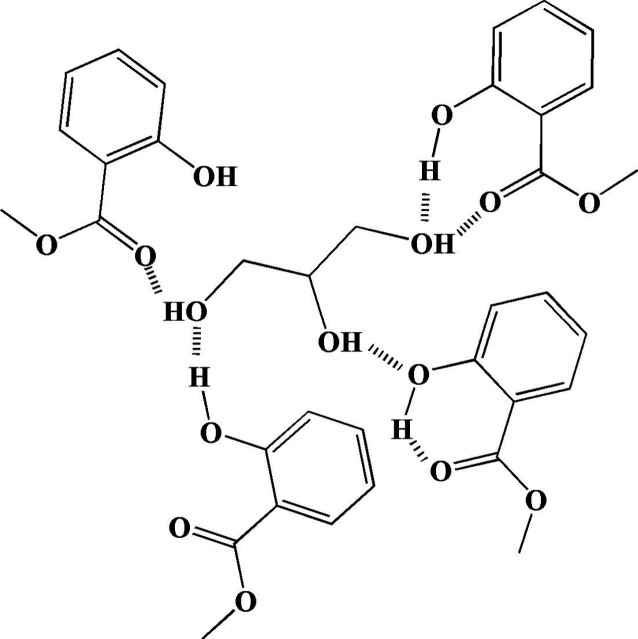
Mechanism of Self-Aggregation of Glycerol and Methyl Salicylate

The pH of both samples (the one containing only alcohol, HS1, and that containing alcohol with methyl salicylate, HS2) was slightly acidic, approximately 5.7 (Table 8), with no significant difference between them (*p* > 0.05). Therefore, the incorporation of methyl salicylate did not significantly alter the final pH of the alcohol gel.

**Table 8:** Characterization of Alcohol Gel in Hand Sanitizer with (HS2) and without (HS1) Methyl Salicylate

parameters	HS1	HS2
pH	5.70 ± 0.01	5.70 ± 0.05
relative density (g/cm^3^)	0.882	0.884
apparent viscosity (cP)	27,180 ± 60	27,080 ± 35
*in vitro* spreadability (mm^2^)	1837.2 ± 191.9	1686.4 ± 111.7

The relative densities of the products ranged from 0.882 to 0.884 g/cm^3^, with minimal variation following the addition of methyl salicylate. The dissolution of methyl salicylate in the gel vehicle did not disrupt the hydrogen bonds between hydroxyethylcellulose and water; therefore, the viscoelasticity of the formulation was not affected. Consequently, the relative density remained essentially unchanged.

Both alcohol gels had a high apparent viscosity, exceeding 20.000 cP, with no statistically significant difference between them (*p* > 0.05). These viscosities were well above the 8.000 cP threshold recommended by ANVISA (2002).^41,42^ The high viscosity observed can be attributed to the composition of the formulation, particularly the concentration of the polymer. Whereas the presence of ethanol typically results in a lower viscosity, the polymer concentration was adequate to counteract this effect. A higher viscosity contributes to improved adhesion, reduced dispersibility, and a prolonged drying time.

Finally, the *in vitro* spreadability test evaluated the ability of a formulation to distribute evenly over the skin. The addition of methyl salicylate did not significantly affect the spreadability of the gel. Although a slight decrease from 1837 to 1686 cP was observed, the difference was not statistically significant (*p* > 0.05). Despite the high viscosity, the formulation maintained a good spreadability (Table 8).

### 3.4 Methods for the Determination of the Antibacterial Activity

Bacterial suspensions were prepared according to CLSI M7-A10 (2015) from 24 h BHI cultures (Himedia; Mumbai, India), adjusted to the 0.5 McFarland standard (∼1.0 × 10^8^ CFU/mL) in sterile saline and diluted in Mueller Hinton Broth (MHB) to 5.0 × 10^6^ CFU/mL. The minimum inhibitory concentration (MIC) was determined in 96-well plates (Global) containing 50 μL of test solutions (methyl salicylate, hand sanitizer gel, or hand sanitizer gel with methyl salicylate) and 50 μL of bacterial suspension. Controls included MHB only, chloramphenicol (30 μg/mL) as the standard, and MHB with the bacterial suspension (growth control). Plates were incubated for 24 h at 37 °C in a bacteriological incubator (Solab; Piracicaba, São Paulo).

Bacterial viability was assessed using the resazurin assay (Alamar Blue dye), as described by Travnickova et al.^43^ An aliquot of 25 μL of 0.01% resazurin solution was added to each well, followed by incubation for 4 h at 37 °C. Antibacterial activity was indicated by wells that remained blue, whereas the development of a pink color denoted bacterial viability.

All of the experiments were performed in triplicate using the strains: Gram-positive bacteria *Enterococcus faecalis* (ATCC 19433), *Streptococcus agalactiae* (ATCC 29313), *Staphylococcus aureus* (ATCC 29313), and the Gram-negative bacterium *Escherichia coli* (ATCC 25922).

An MIC of 1 g/mL (Figure 6) was observed for all the concoctions tested (hand sanitizer gel only, methyl salicylate only, and hand sanitizer gel with methyl salicylate), regardless of the bacterial strain. This result indicates that the hand sanitizer gel, at high concentrations without dilution, is effective against both Gram-positive and Gram-negative bacteria. Similarly, methyl salicylate, either alone or combined with the hand sanitizer, also maintained this activity.

**Figure 6:**
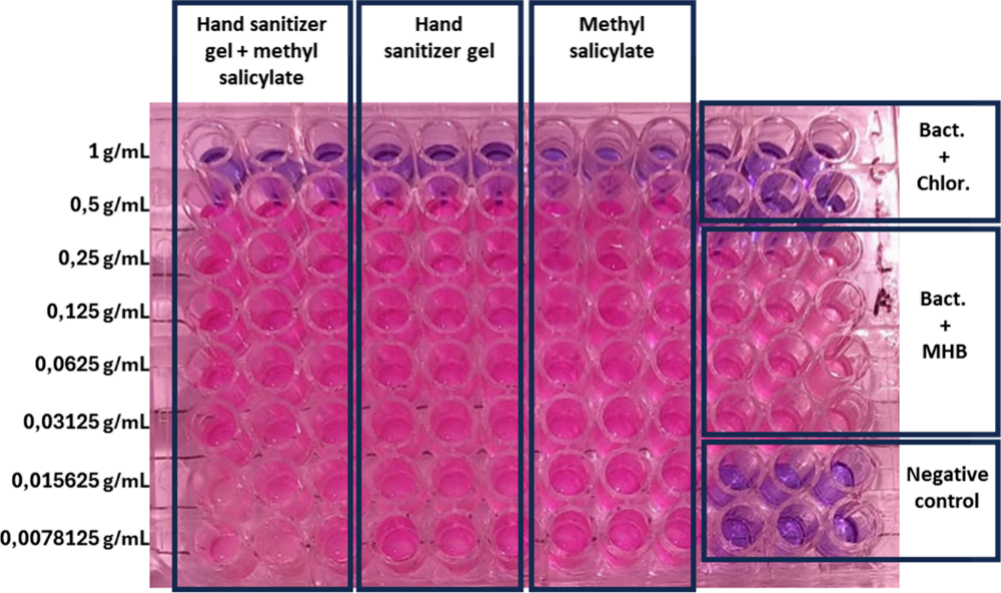
Inhibitory activity of the compounds against *Streptococcus agalactiae* . Bac + chlor. = positive control (chloramphenicol); Bac + medium = growth control; negative control = MHB only.

Antimicrobial efficacy of a hand sanitizer depends not only on the final alcohol concentration, ideally 70 to 80% w/w, but also on skin contact time, which is influenced by viscosity and flow behavior, and on the rate of evaporation of the ethanol.^47^ The addition of other compounds should not reduce the alcohol content below the effective threshold, impair ethanol diffusion, or excessively alter formulation rheology.

Glycerol slightly increases the viscosity while reducing water activity. As a humectant, it decreases ethanol evaporation, prolonging its action on the skin and potentially reducing ethanol-induced irritation, thereby improving adherence to continuous use.^7^ Methyl salicylate exhibits anti-inflammatory and photoprotective activity and may potentiate the effect of ethanol due to its mild antimicrobial activity. Potential skin irritation is minimized by its use associated with glycerol.^48,50^

## 4 Conclusions

The purification of crude glycerol represents a critical process in the effective management of substantial waste byproducts generated during the transesterification of triglycerides. Crude industrial glycerol donated by the biodiesel industry was treated and purified by adsorption on commercial charcoal. The application of the absorption technique for purifying crude industrial glycerol offers a method to convert crude glycerol into higher-quality materials, reduce environmental impact, support circular resource use, and improve sustainability. In this study, purified glycerol obtained by adsorption, particularly using activated carbon, is highly effective for decolorizing and the removal of impurities (reducing color by over 99%). The yields obtained here (99.428 and 99.624% yield) are higher than those found in the literature using similar techniques.^49^ It is a byproduct of biodiesel production via acid- or base-catalyzed transesterification. The process can be easily adapted to industrial-scale processes, reaching 50 tons/day or more. These methods demonstrate the high degree of scalability, starting with the pretreatment of the crude glycerol, where inorganic salts are precipitated and removed. After an adsorption process (e.g., activated carbon), the glycerol can be processed in large reactors using thermal heating and high pressure to accelerate the filtration of the pretreated material until the product is completely bleached. Purified glycerol was introduced as a humectant in the hand sanitizer formulation for the conversion of glycerol to valuable products. A simple, rapid method for quantifying ethanol in ethanol-based gel hand sanitizers by HS-GC/FID was employed. Even after the COVID-19 pandemic, the demand for ethanol-based hand sanitizers continues to be high, and the method described was shown to furnish excellent results regarding the effectiveness as a hand sanitizer. It also represents an alternative use for crude industrial glycerol being produced on a large scale by the biodiesel industry. The principal critical quality attributes of the hand sanitizer were achieved to guarantee the safety of its use as a dermatological product. Furthermore, the use of methyl salicylate did not change the satisfactory characteristics of the product. The product satisfies the requirements of the WHO that prescribe formulations containing 1.45% glycerol as a humectant to protect HCWs' skin against dryness and dermatitis. We hope these results inspire further multidisciplinary research on purification, refining, and the discovery of new uses for crude glycerol.

## Data Availability

The paper entitled ‘‘Valorization of a Byproduct for Sustainable Formulations: Preparation of an Alcohol Gel Hand Sanitizer Containing Glycerol and Methyl Salicylate” is submitted for your kind consideration. The research data policy and data availability are not shared with anyone before publication.
